# A patient with colitis-associated cancer who developed clinically manifest Crohn’s disease only after surgery

**DOI:** 10.1186/s40792-020-0779-2

**Published:** 2020-04-10

**Authors:** Yoshio Shimizu, Hideya Takaku, Sugiru Paku, Kazuaki Azuma, Toshishige Suzuki, Hiroshi Kashimura, Haruo Ohtani, Nobuhiro Ohkochi

**Affiliations:** 1 Department of Gastrointestinal and Hepato-Biliary-Pancreatic Surgery, Faculty of Medicine, University of Tsukuba, 1-1-1 Tennodai, Tsukuba, 305-8575 Japan; 2 Departments of Surgery, Mito Saiseikai General Hospital, 3-3-10 Futabadai, Mito, 311-4145 Japan; 3 Departments of Gastroenterology, Mito Saiseikai General Hospital, 3-3-10 Futabadai, Mito, 311-4145 Japan; 4 Departments of Pathology, Mito Saiseikai General Hospital, 3-3-10 Futabadai, Mito, 311-4145 Japan; 5 Department of Surgery, Mito-chuo Hospital, 1136-1 Rokutanda chou, Mito, 311-1135 Japan

**Keywords:** Colitis-associated cancer, clinically latent IBD, dysplasia

## Abstract

**Background:**

Patients with prolonged inflammatory bowel disease have a greater risk of colorectal cancer, known as colitis-associated cancer. Here we describe an unusual case of colitis-associated cancer.

**Case presentation:**

The subject is a 41-year-old male who has not presented digestive symptoms and has an appreciable medical history. He consulted a nearby doctor with left flank pain. A colonoscopy revealed a lateral spreading tumor (granular-type) in his descending colon. With a clinical diagnosis of cancer, D3 left hemicolectomy was performed and a small intestine stoma was constructed. The pathological diagnosis of the tumor was mucinous adenocarcinoma, pT4a(SE), pN2a, which was associated with dysplasia in the surface area. Post-operative ileus was prolonged and the endoscopic examination revealed longitudinal ulcers in the ileum. These ulcers responded quite well to the administration of infliximab, confirming the final diagnosis of Crohn’s disease. Pathological re-examination revealed that the tumor was dysplasia-associated type, and another dysplasia was confirmed near the tumor. Furthermore, mural scars and sporadic lymphoid aggregates were noted in the colon tissues, which suggested pre-existing Crohn’s disease. The patient died of peritoneal dissemination of cancer on day 207 after surgery.

**Conclusion:**

The present case was diagnosed as colitis-associated cancer with clinically latent Crohn’s disease, who developed clinically manifest Crohn’s disease only after surgery. Our review of literature revealed no cases comparable to ours.

## Background

The cause of Crohn’s disease has not been elucidated, and it is regarded as a multifactorial disease for which environmental factors, genetic factors, and immunological abnormality are compounded [1]. The incidence of Crohn’s disease has been increasing in recent years in Japan, with the number of patients exceeding 40,000 considering the number of certifications of Crohn’s Disease Medical Care Recipient, 2014. Prolonged morbidity of Crohn’s disease increases the risk of colorectal cancer. Thus, the number of patients with Crohn’s disease-associated colorectal cancer is increasing [2]. However, there are few reports of asymptomatic Crohn’s disease-associated colorectal cancer. Here, we describe an unusual case of colitis-associated cancer who developed clinically manifest Crohn’s disease only after the resection of cancer.

## Case presentation

The patient is a 41-year-old male. His chief complaint is left flank pain. He has no medical history/family history.

### Clinical history

The subject consulted a nearby doctor with left stomachache and fever as chief complaints in 1 month prior to the surgery. Diverticulitis of the descending colon splenic flexure was doubted in CT and the subject was hospitalized. A colonoscopy revealed an irregular flat elevated lesion on the splenic flexure. The subject was transferred to our hospital for examination and medical treatment.

### Physical examination

The height is 175 cm, body weight 54.6 kg, pulse 66 beats/m, body temperature 36.3, and blood pressure 114/76 mmHg. The abdomen was slightly distended, and mild left stomachache was recognized. The stool frequency was 1 time/day.

### Test result at the time of hospitalization

In blood biochemistry examination, values were as high as 22.5 ng/ml in CEA and 81.0 U/ml in CA19-9 tumor markers.

### Abdominal contrast-enhanced CT

**Fig. 1:**
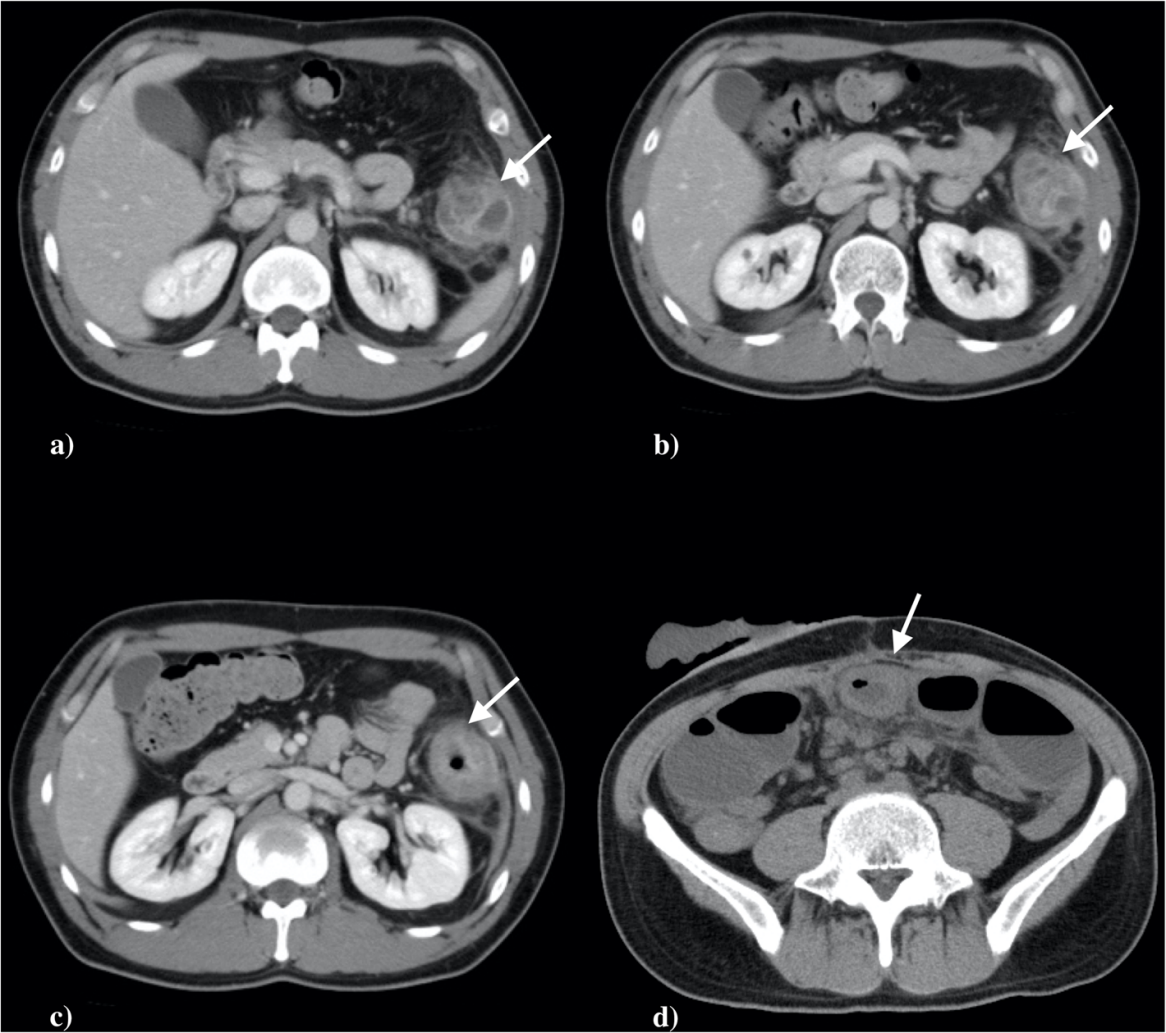
**a**–**d** Enhanced CT scan of abdomen showed highly enhanced thickened wall of the colon in the splenic flexure (arrow)

Significant hyperplasia (4 cm) of the colon mucous membrane was observed on the descending colon splenic flexure and it was accompanied by elevation of surrounding adipose tissue density. The surrounding lymph nodes swelled up to around 5 mm. Since strong hyperplasia was seen in the mucous membrane, it was thought as reactive enlargement accompanied by inflammation. The finding that indicates distant metastasis was not seen in the liver and lung (Fig. 1).

### Colonoscopy

**Fig. 2:**
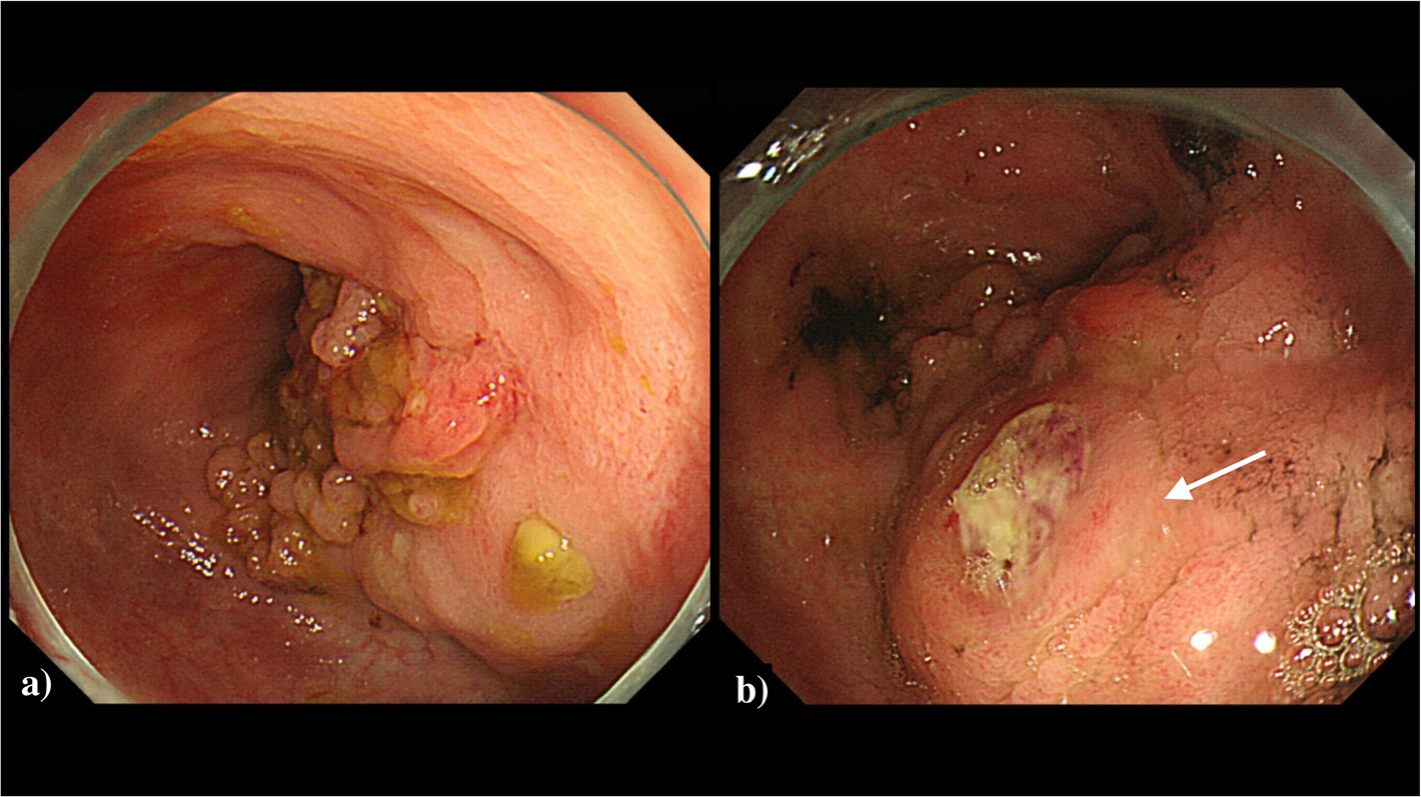
**a** Preoperative colonoscopy showed a group of a lateral spreading tumor of granular type of descending colon. **b** Second colonoscopic examination revealed the biopsy site of tumor (white arrow) was abnormally edematous

A semicircular, lateral spreading tumor of granular-type (i.e., an aggregate of IIa-like tumors) was observed in the descending colon splenic flexure (Fig. 2a).

### Gastroscopy

Notable abnormality such as irregular ulcer and scar or bamboo joint-formed appearance was not recognized.

### Progress after the hospital transfer

Biopsy was performed twice for the lateral spreading tumor of granular-type of descending colon. The tumor was diagnosed pathologically as a tubular adenoma without evidence of malignancy. However, we judged that the lesion in the descending colon was a cancer based on the elevated CEA level and CT findings. We also considered the possibility of coexistence of inflammatory lesion near the tumor because of an edematous change of the mucosa (Fig. 2b). Therefore, it was determined to place a covering stoma 20 cm proximal from the terminal ileum at the time of surgery.

### Surgery

D3 left hemicolectomy and loop type stoma construction were performed. No abnormality was confirmed in the small intestine such as thickness, fistula, mass, and adhesion to abdominal wall during the operation.

### Histopathological examination (including results of re-examination)

**Fig. 3:**
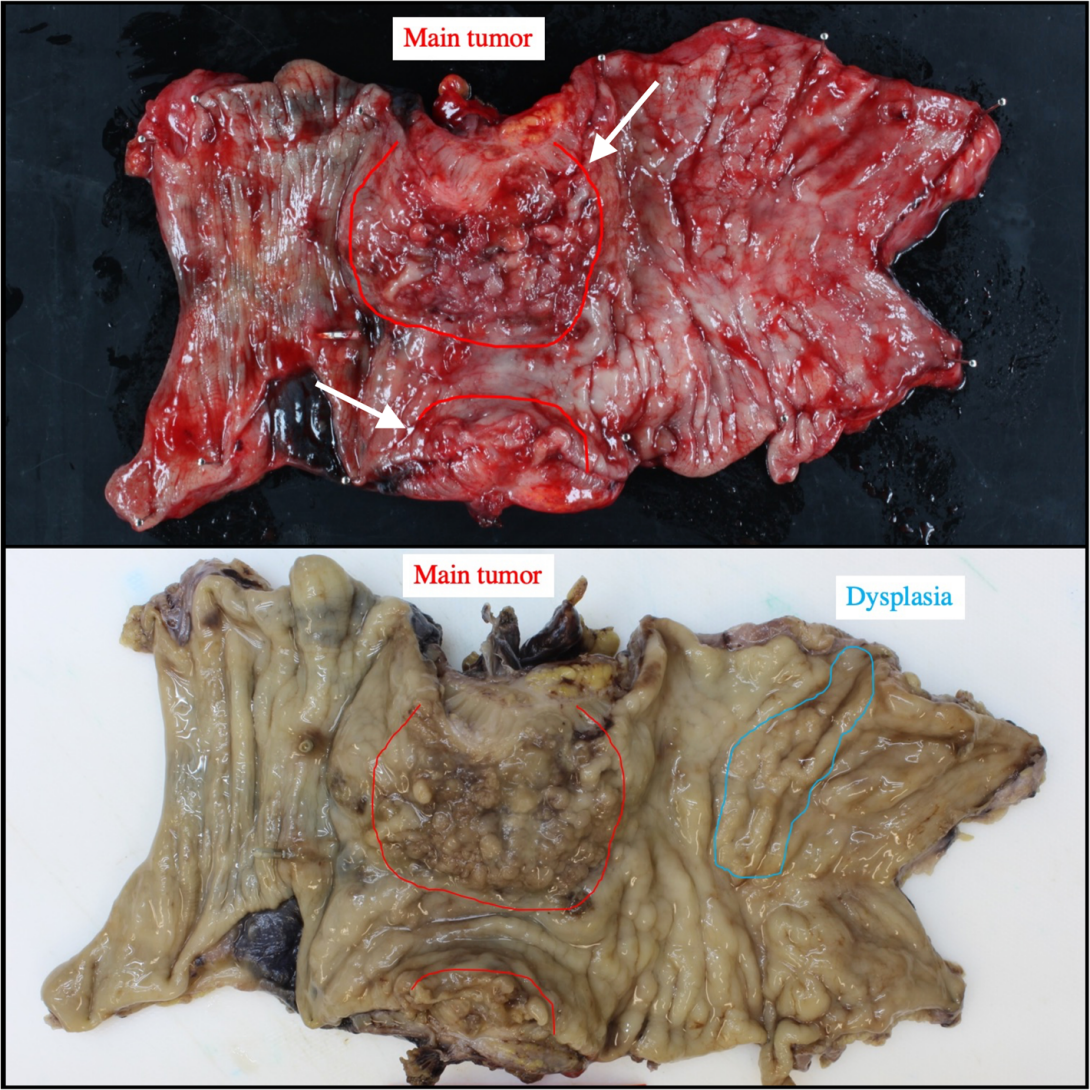
**a** The resected specimen of the colon showed the main tumor was a lateral spreading tumor (granular type), 50 × 48 mm in size, in the transverse colon (arrow). **b** Blue circle indicates another dysplasia, 5.2 × 1.8 cm in suze, near the tumor

**Fig. 4:**
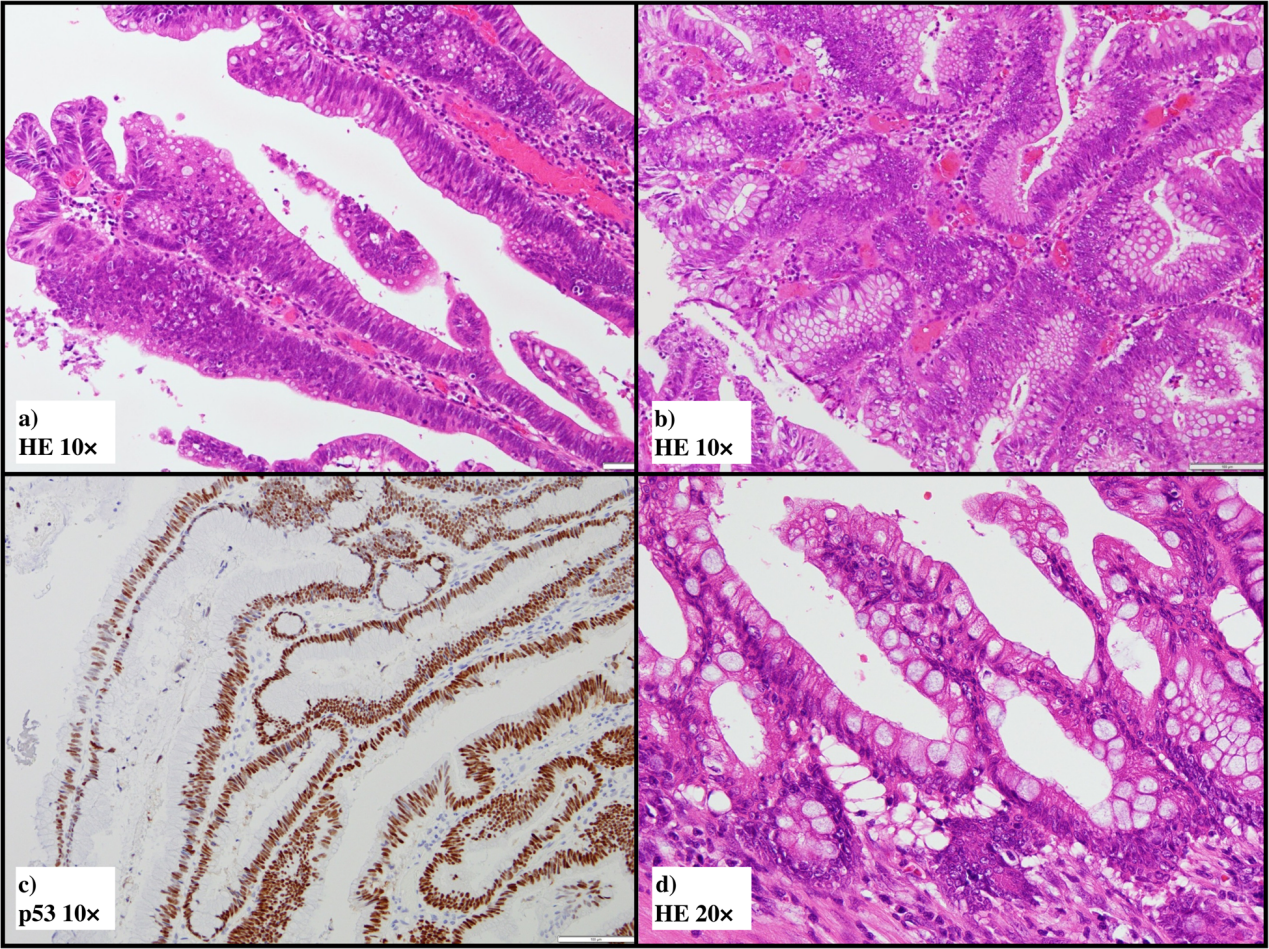
Microscopic findings of the main tumor (**a** HE ×10, **b** HE ×10, c p53 ×10, d HE ×20). The surface area of the main tumor showed proliferation of atypical gland of either villous (**a**) or tubular pattern (**b**) in the mucosa. **c** Immunohistochemistry reveals that nearly 100% atypical glands in the surface area overexpression of p53. These observations confirmed the diagnosis of dysplasia in the surface area. **d** Atypical gland indistinguishable from dysplasia invaded the muscularis propria (well-differentiated adenocarcinoma)

**Fig. 5:**
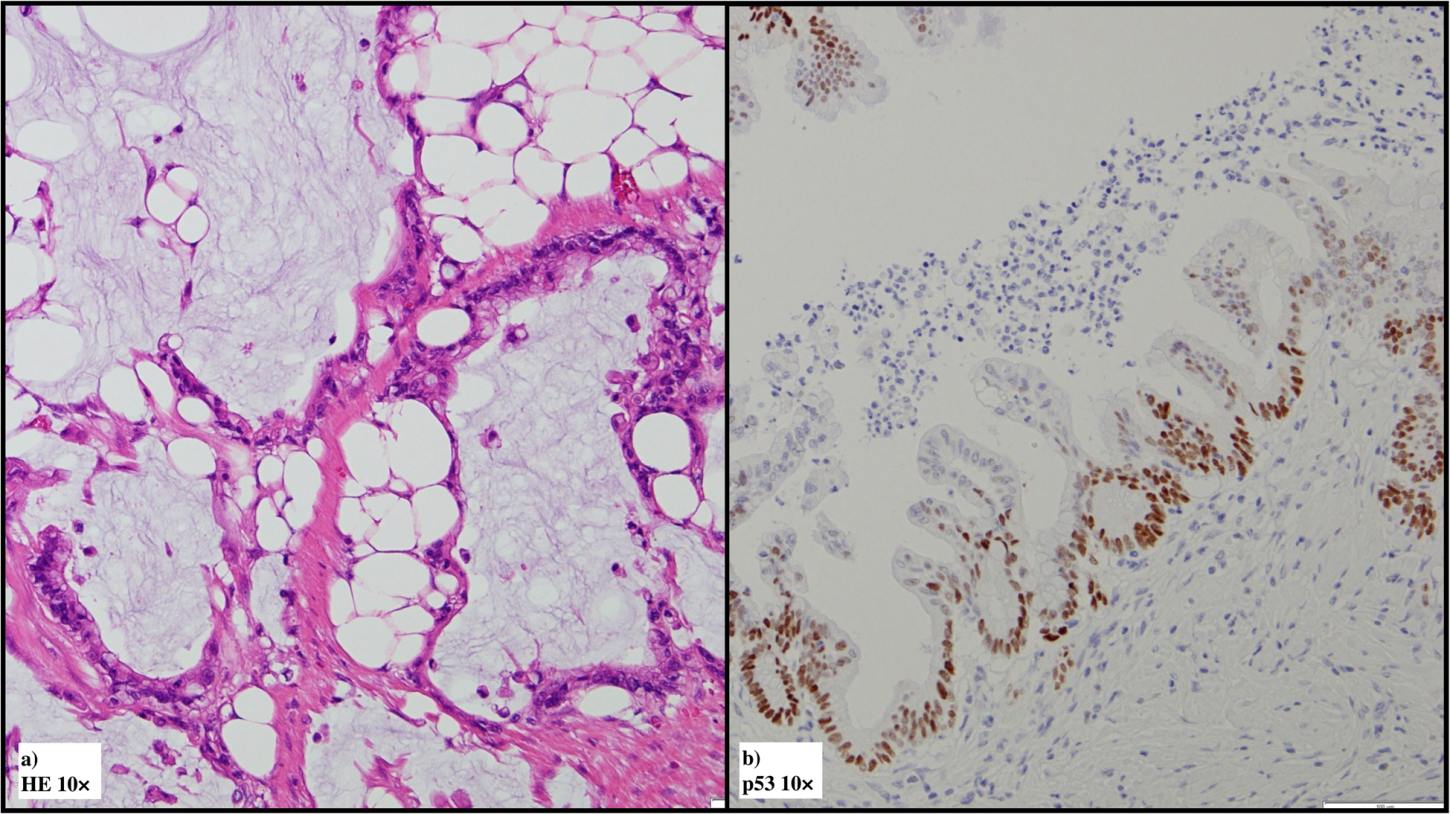
Microscopic findings of the deep area of main tumor (**a** HE ×20, **b** HE ×10, **c** p53 ×10, **d** HE ×20). **a** In deeper area, tumor cells secrete large amount of mucin (mucinous adenocarcinoma). **b** p53 was positive in 50% of tumor cells in mucinous carcinoma

**Fig. 6:**
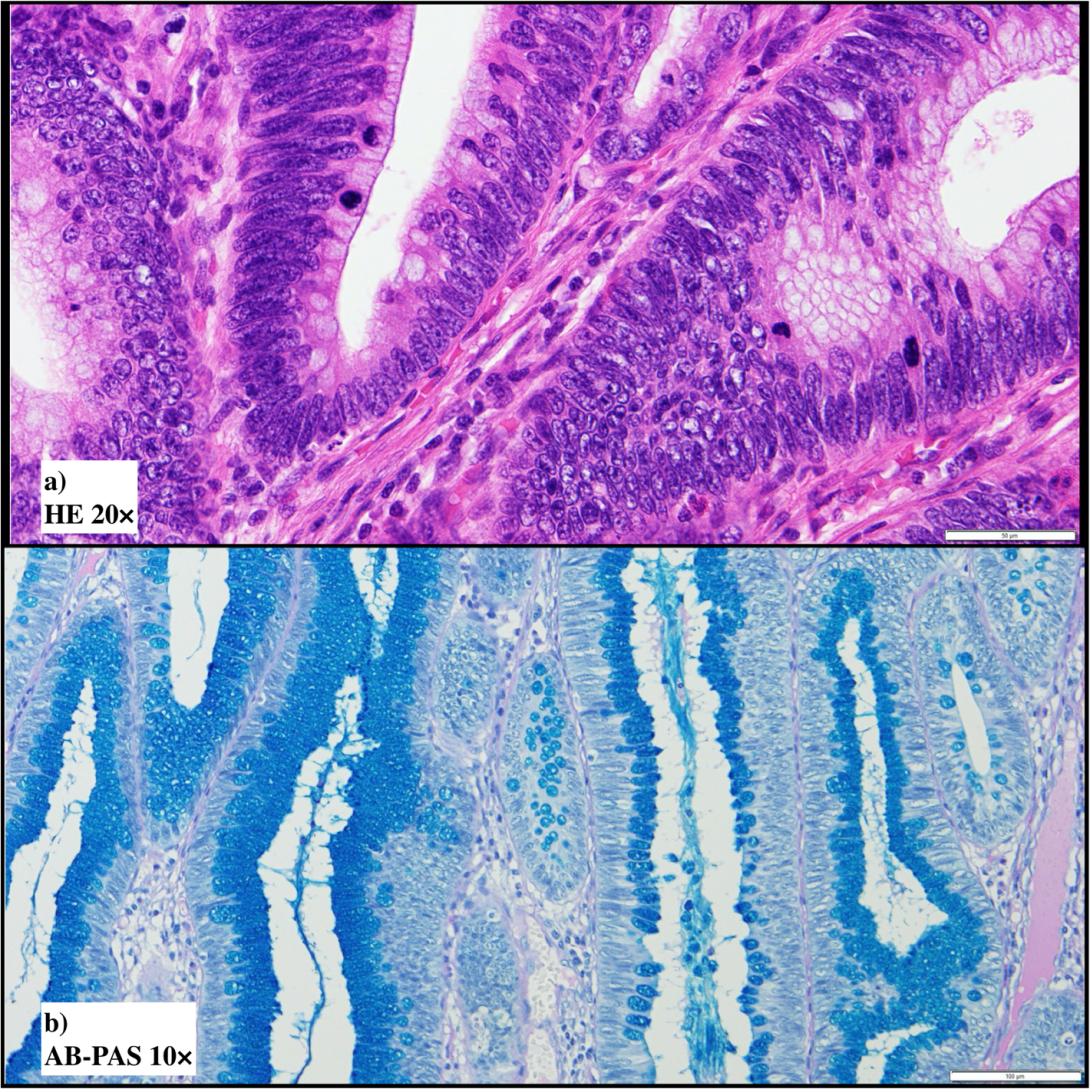
Microscopic findings of the dysplasia indicated by blue line in Fig. 3b (**a** HE ×20, **b** alcian blue—PAS ×10). **a**, **b** Low- and high-grade dysplasia in this lesion had alcian blue-positive mucin in the supra-nuclear area

**Fig. 7:**
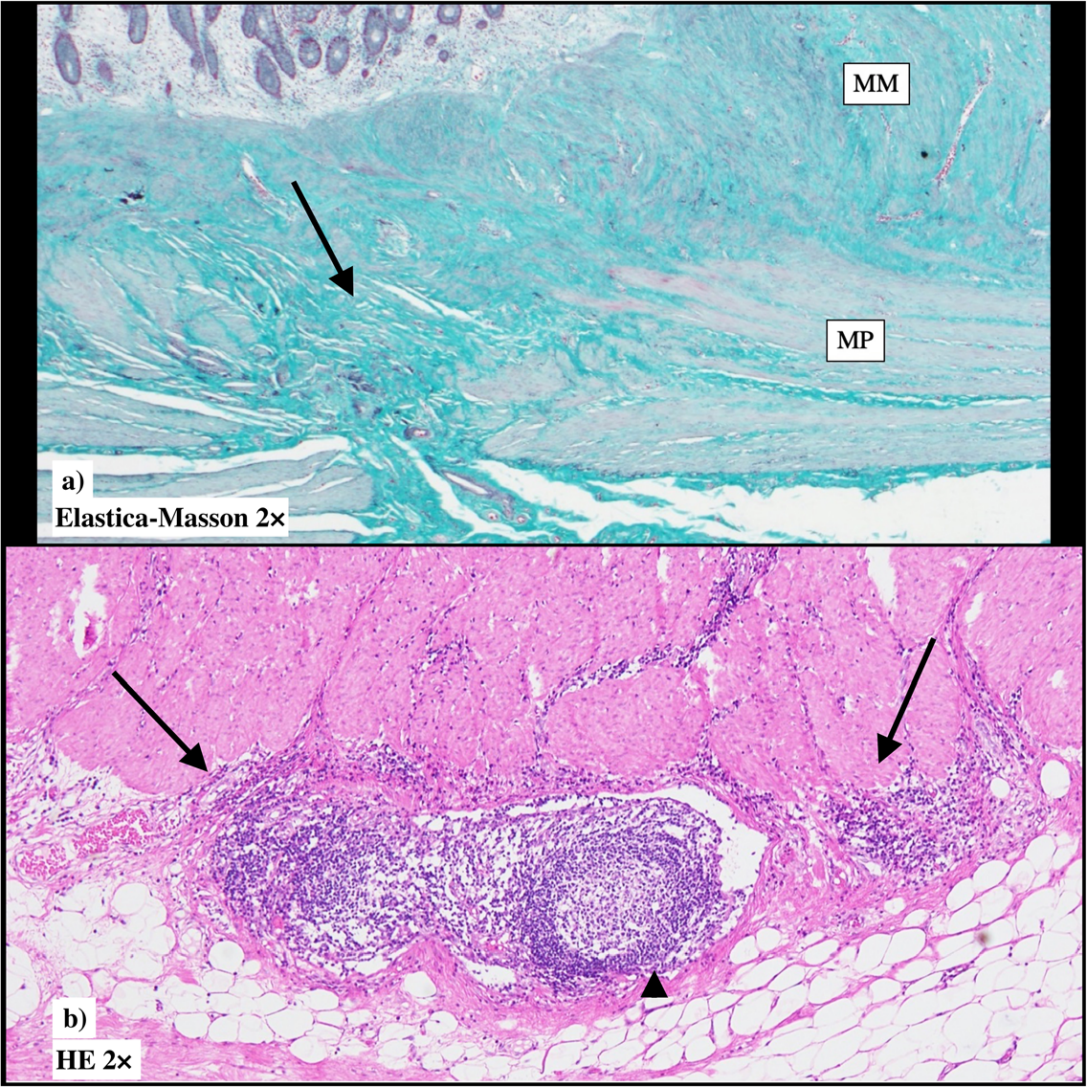
Microscopic findings in the colon tissue near the main tumor suggesting pre-existing latent Crohn’s disease. **a** Scar formation (green) that penetrates the muscularis propria (arrow). **b** Lymphoid aggregate (arrows) and lymphoid follicles in the subserosa (arrow head)

Gross examination confirmed that the main tumor was a laterally spreading tumor (granular-type) in the descending colon (Fig. 3a, b). No gross findings were recognized that supported Crohn’s disease, e.g., longitudinal ulcers, their scars, or cobblestone appearance. Histopathologically, the surface of the main tumor was composed of uniform proliferation of atypical glands (either tubular or villous in configuration) and containing both low- and high-grade areas (Fig. 4a, b). This lesion stained diffusely positive for p53 by immunohistochemistry (Fig. 4c). Therefore, we diagnosed these atypical glands in the surface area this as dysplasia, and this lesion continuously invaded the muscularis propria (Fig. 4d). In deeper areas of the main tumor, deposition of extracellular mucin was observed leading the diagnosis of mucinous adenocarcinoma (Fig. 5a). p53 was positive in 50% of tumor cells in mucinous carcinoma (Fig. 5b). The final diagnosis was mucinous adenocarcinoma associated with dysplasia, in the descending colon, type 5, 50 × 48 mm in size, pT4a (SE) pN2a (4/28), Ly1, V1, cM0, pStage IIIC (UICC TNM classification, 8th ed., and Japanese classification of colorectal carcinoma, 9th ed) [3]. Furthermore, another dysplasia, not continuous from the main tumor, was confirmed by multiple sampling analyses in the resected specimen approximately 5.2 × 1.8 cm in size (indicated by blue line in Figs. 3b and 6). This dysplasia was not associated with adenocarcinoma (i.e., completely confined in the mucosa). We further obtained the following findings that supported pre-existing transmural chronic inflammation (e.g., Crohn’s disease) as follows: (a) irregular thickening of the muscularis mucosae, (b) fibrosis in the submucosa and muscularis propria, and (c) sporadic formation of lymphoid aggregates in the submucosa and subserosa in the resected colon specimen (Fig. 7). Taken together, the histopathological features of main tumor were consistent with colitis-associated cancer.

### Postoperative course

**Fig. 8:**
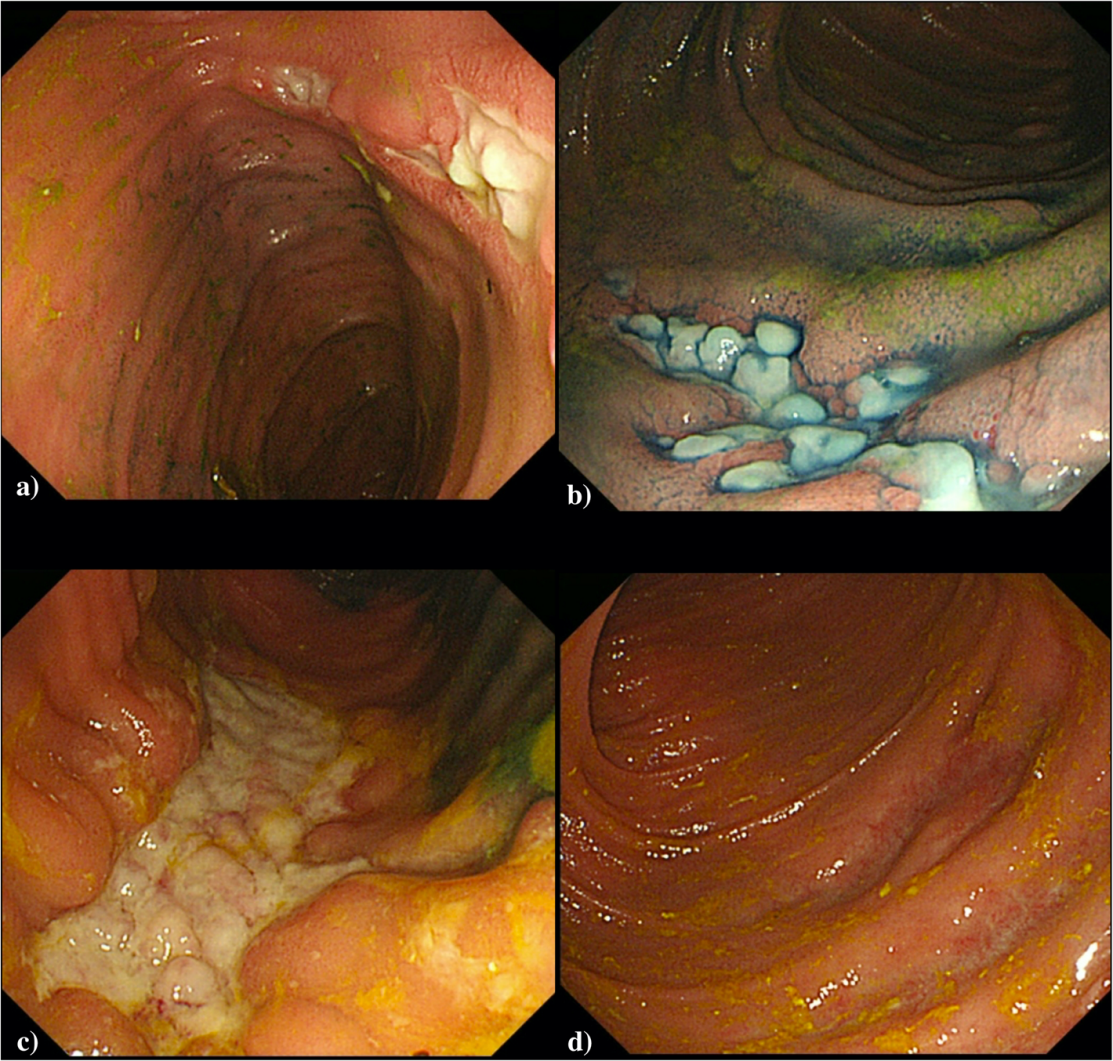
**a**, **b** Postoperative endoscopic examination showed annular ulcers in the ileum in POD 55th. **c** A longitudinal ulcers in the ileum in POD 77th. **d** After administration of infliximab, ulcers healed rapidly in POD103th

The ileus state was prolonged for over 40 days after the surgery. On day 55 endoscopies were performed to newly find orbicular ulcers in the terminal ileum (Fig. 8a, b). We excluded the possibility of tuberculosis or cytomegalovirus by an interferon-gamma release assay and serum antibody assay. Re-examination of endoscopy on day 77 revealed the development of longitudinal ulcers in the ileum (Fig. 8c). With a clinical diagnosis of Crohn’s disease, the patient received infliximab administration, and the lesions of small intestine ameliorated significantly on day 103. Furthermore, ileus was improved and therefore the subject was discharged on day 112 (Fig. 8d). Seventy-three days after the discharge, CEA suddenly was elevated and intestinal edema, and ascitic fluid appeared in abdominal contrast-enhanced CT. The subject was diagnosed as ileus attributable to peritoneal dissemination. Overall status gradually worsened and he passed away on day 207. The final cause of death was judged as peritoneal dissemination.

## Discussion

Prevalence and incidence rates of Crohn’s disease in Asian countries have been increasing similar to western countries [4]. However, there is a difference in cancer site between Japanese and western countries [5, 6]. Compared to western countries, the incidence of anorectal cancer is higher than colon cancer. Additionally, it is frequent of carcinogenesis in fistula of the anal lesion. In Japan, as same as western countries, the risk factor for the colorectal cancer is longer duration of disease [7].

The most important issue of the present case is the diagnosis of Crohn’s disease. Clinically manifest findings and symptoms were noted only after the surgery. On day 77, longitudinal ulcers were detected by the endoscopy. The possibility of tuberculosis or cytomegalovirus infection was excluded by laboratory data as mentioned in the case presentation. Drastic improvement of ulcers by infliximab administration finally confirmed Crohn’s disease. Retrospective analyses also confirmed the following findings that suggest the presence of Crohn’s disease near cancer, e.g., mucosal edematous changes by endoscopy before the surgery and histopathological findings, i.e., irregular thickening of the muscularis mucosae, fibrosis in the submucosa and muscularis propria, and sporadic formation of lymphoid aggregates in the submucosa and subserosa in the colon tissue. These suggest the presence of clinically latent Crohn’s disease before surgery.

The diagnosis of colitis-associated cancer included the followings: association of dysplasia in the surface area of the main tumor and detection of another dysplasia near the main tumor. Taken together, we could be able to diagnose the present case as colitis-associated cancer with clinically latent Crohn’s disease who developed clinically manifest Crohn’s disease after surgery.

There are only a few reports of clinically latent Crohn’s disease in a western country and Japan [8].

Rodríguez-Lago I reported that retrospective analysis of 31005 colonoscopies revealed previously asymptomatic 110 patients were diagnosed as ulcerative colitis [9]. Similar study was reported in Japan. Retrospective analysis of 236,000 colonoscopies in Japan revealed previously asymptomatic 12 patients were diagnosis as ulcerative colitis [10]. However, there was no report about clinically latent Crohn’s disease with colon cancer. We searched for similar colon cancer case reports in Japan with Crohn’s disease whose disease duration within 1 year, excluding rectal anal cancer. Eight cases including our case were reported. Males accounted for 75% and their average age was 42.3 years old. However, other cases had an abdominal symptom. The similar case with asymptomatic Crohn’s disease with ileal cancer was reported [11]. The case had an occult tumor in ileocecal mass without abdominal symptoms. However, the findings of abdominal CT showed there was a great mass with surrounding effusion. The dissociation of abdominal symptoms and CT findings was very similar to our case. Thus, if there was a dissociation between abdominal symptoms and the CT findings, there might be a malignant tumor in the site.

We presented here an exceptional case of colitis-associated cancer who developed clinically manifest Crohn’s disease only after the surgery. To identify such cases, careful clinical and histopathological analyses would be important.

## Conclusion

In summary, we described a very rare case of a colitis-associated cancer with clinically latent Crohn’s disease. If the patient with no abdominal symptoms showed abdominal mass with surrounding effusion in abdominal CT, it is important to recognize that there might be the malignancy in the mass.

The dissociation abdominal symptoms and CT findings might be the key findings of the clinically latent Crohn’s disease with colon cancer. Surgeons should examine such case carefully and observe the intestine attentively with suspicion of IBD.

## Data Availability

Not applicable

## Abbreviations

CA19-9: Carbohydrate Antigen 19-9
CEA: CarcinoEmbryonic Antigen
CT: Computed Tomography
IBD: Inflammatory Bowel Disease
MM: Muscularis Mucosae
MP: Muscularis propria
